# 26SCS-Loaded SilMA/Col Composite Sponge with Well-Arranged Layers Promotes Angiogenesis-Based Diabetic Wound Repair by Mediating Macrophage Inflammatory Response

**DOI:** 10.3390/molecules29081832

**Published:** 2024-04-17

**Authors:** Pin Luo, Wei Liu, Zhangyao Ye, Yuyu Zhang, Zekun Zhang, Jing Yi, Rong Zeng, Shenyu Yang, Mei Tu

**Affiliations:** 1 College of Chemistry and Materials Science, Jinan University, Huangpu Road 601, Guangzhou 510632, China; pinkew@stu2021.jnu.edu.cn (P.L.); rw1107@stu2022.jnu.edu.cn (W.L.); yezhangyao@stu2021.jnu.edu.cn (Z.Y.); nayu13@stu.2021.jnu.edu.cn (Y.Z.); kk9781@stu2021.jnu.edu.cn (Z.Z.); faye254330749@petalmall.com (J.Y.); zengronga@jnu.edu.cn (R.Z.); yangsy@jnu.edu.cn (S.Y.); 2 Engineering Research Center of Artificial Organs and Materials, Ministry of Education, Jinan University, Guangzhou 510632, China

**Keywords:** sulfated chitosan, macrophages, inflammatory, angiogenesis, diabetic wound healing

## Abstract

Diabetic wound healing is a significant clinical challenge because abnormal immune cells in the wound cause chronic inflammation and impair tissue regeneration. Therefore, regulating the behavior and function of macrophages may be conducive to improving treatment outcomes in diabetic wounds. Herein, sulfated chitosan (26SCS)-containing composite sponges (26SCS-SilMA/Col-330) with well-arranged layers and high porosity were constructed based on collagen and silk fibroin, aiming to induce an appropriate inflammatory response and promote angiogenesis. The results indicated that the ordered topological structure of composite sponges could trigger the pro-inflammatory response of Mφs in the early stage, and rapid release of 26SCS in the early and middle stages (within the concentration range of 1–3 mg/mL) induced a positive inflammatory response; initiated the pro-inflammatory reaction of Mφs within 3 days; shifted M1 Mφs to the M2 phenotype within 3–7 days; and significantly up-regulated the expression of two typical angiogenic growth factors, namely *VEGF* and *PDGF-BB*, on day 7, leading to rapid HUVEC migration and angiogenesis. In vivo data also demonstrated that on the 14th day after surgery, the 26SCS-SilMA/Col-330-implanted areas exhibited less inflammation, faster re-epithelialization, more abundant collagen deposition and a greater number of blood vessels in the skin tissue. The composite sponges with higher 26SCS contents (the (5.0) 26SCS-SilMA/Col-330 and the (7.5) 26SCS-SilMA/Col-330) could better orchestrate the phenotype and function of Mφs and facilitate wound healing. These findings highlight that the 26SCS-SilMA/Col-330 sponges developed in this work might have great potential as a novel dressing for the treatment of diabetic wounds.

## 1. Introduction

Diabetic patients suffer from chronic non-healing wounds [1], mainly due to tissue loss and inadequate blood flow to the wound bed [2,3]. Therefore, the reconstruction of the micro-vascular network becomes a significant challenge for the tissue repair of diabetic wounds. A large number of experimental and clinical studies have shown that the healing of diabetic wounds does not follow the normal process of wound healing seen in acute wounds due to the complex physiological micro-environment in chronic wounds, and the repair effect achieved by using conventional wound dressings is very limited. Therefore, new technologies are being translated from the test bench to clinical practice using bio-active materials and synthetic bio-polymers, aiming to provide new strategies for diabetic wound healing.

At present, the dressings used for the treatment of diabetic wounds mainly include hydrogels, electrospun nanofibers, bio-macromolecule scaffolds/sponges and bio-active glass wound dressings [4,5], all of which have shown the potential to enhance diabetic wound healing. For example, Lv et al. reported a silicate-containing poly(caprolactone)/gelatin nanofibrous composite scaffold for diabetic wounds and suggested that the composite scaffold had a synergetic effect, improving the efficiency of diabetic wound healing [6]. Pietramaggiori et al. fabricated a poly N-acetylglucosamine hydrogel matrix system for the treatment of diabetic foot ulcers [7,8,9]. Yu and co-workers prepared a curcumin-loaded in situ injectable hydrogel based on sodium alginate and chitosan. Nano-curcumin in a hydrogel was able to leach slowly to promote fibroblast proliferation, collagen generation and capillary formation, leading to diabetic wound healing [10,11]. Yang et al. [12] utilized electrospinning technology to prepare nanofiber pad silk fibroin dressings containing epidermal growth factor (EGF) and basic fibroblast growth factor (bFGF). The results indicated that reconstructed nanofiber pads could support and enhance cell migration to the wound bed in high-glucose environments, then promote wound healing [13]. In addition, bio-macromolecules and their complexes based on carboxymethyl cellulose, silk fibroin, chitosan and collagen have also been used as wound dressings and show great promise in inducing tissue regeneration in diabetic wound healing [6].

Silk fibroin is a natural amino acid polymer with a composition and structure similar to that of a natural extracellular matrix. It can penetrate the surface of wounds and maintain a gentle, moist environment to promote wound healing [14]. Collagen-based skin dressings have also been reported to have a rapid and effective therapeutic effect on diabetic wounds [15] because collagen and its degradation products and metabolic derivatives can attract fibroblasts to wounds and enhance skin repair [16,17]. For example, collagen dressings combined with silver and sodium alginate have achieved great efficacy in the treatment of diabetes wounds [18]. Han et al. constructed a new type of collagen-based dermal scaffold coated with silver nanoparticles (NAg). The results demonstrated that NAg-containing scaffolds exerted bactericidal and anti-inflammatory effects and promoted wound healing by regulating fibroblast migration and macrophage activation [19]. Moreover, collagen can be used in the form of a hydrogel, freeze-dried sponge, membrane, inner membrane or nanofibers generated by electrospinning [20], showing advantages in various wound-repair scenarios [15].

In recent years, with the continuous growth and evolution of the number of tissue repair models used in research, our understanding of tissue repair and our ability to combine this knowledge with the pathogenesis of related diseases have also been expanded. Nowadays, it is widely believed that the immune system plays a crucial role in tissue healing. Immune cells directly affect the host response at the site of injury, as well as the activity of tissue-specific cell populations and recruited and tissue-resident stem cells. In fact, the immune system has been shown to have positive and negative regulatory effects on tissue repair processes, leading to effective tissue regeneration or fibrosis and scar formation, respectively [21,22]. In the early stage of inflammation [23], primitive and inactive M0 macrophages (Mφs) are primarily activated to the pro-inflammatory M1 phenotype, which initiates an immune response and engulfs micro-organisms, pathogens, abnormal cells and cell fragments. Additional white blood cells are also recruited and produce chemokines [24]. During the later healing process, M0 macrophages mainly transform into the M2 phenotype with anti-inflammatory effects and can release various anti-inflammatory cytokines to assist in wound healing [25]. The biological functions of M2 macrophages also include promoting fibroblast proliferation; collagen remodeling; neovascularization; and the expression of anti-inflammatory mediators such as *IL-1R* antagonists, *IL-10*, transforming growth factor β (*TGF-β*) and vascular endothelial growth factor (*VEGF*) [26]. However, unlike normal acute wounds, there is a large number of M1 phenotype macrophages in chronic wounds of diabetes, and these macrophages excessively produce related pro-inflammatory cytokines such as tumor necrosis factor α (*TNF-α*), interleukin-6 and *IL-1β*. The increase in these key pro-inflammatory cytokines leads to a prolonged inflammatory phase and delayed wound healing [27]. Therefore, in view of the special micro-environment of diabetes wounds, transforming macrophages from the M1 to M2 phenotype and promoting the secretion of anti-inflammatory cytokines may be effective ways to regulate the inflammatory process of diabetic wounds. In addition, in the process of immune regulation, the characteristics of implanted materials inducing the aggregation of immune cells in vivo [28] have been shown to play a direct role in angiogenesis [29,30], which can improve the reconstruction of the micro-vascular network during ischemia of diabetes symptoms and promote the repair of wound tissue.

Heparin (Hep) is a highly sulfated glycosaminoglycan (GAG) that interacts with many proteins to regulate physiological and pathological processes [31]. The combination of heparin and angiogenic growth factor has been shown to have significant therapeutic effects in the treatment of ischemic injury [32,33]. However, heparin has limitations in clinical application due to its anticoagulant properties [34,35]. Sulfated polysaccharides (such as sulfated chitosan (SCS)) are a kind of heparin-like anionic polysaccharide with a higher degree of sulfation and a similar structure to that of heparin. SCS has shown promise for a wide range of applications because of its capacity to bind to protein growth factors [36,37] and regulate the biological activity of growth factors [37]. Liu et al. developed a high-affinity SCS for vascular endothelial growth factor (*VEGF*) and found that it could effectively promote angiogenesis in the presence of extremely low concentrations of exogenous *VEGF* [38]. The release of SCS from an SCS-coated gelatin sponge (SCS GS) was able to effectively stimulate M1-M2 polarization of Mφs and induce endothelial cell migration and capillary formation, thus promoting angiogenesis with the help of very low doses of *VEGF* [38].

In this work, we develop a lamellar-oriented collagen/silk fibroin porous sponge loaded with SCS, aiming at guiding the polarization of Mφs from M1 to M2 and inducing efficient angiogenesis in diabetic wounds. The excellent bio-compatibility and skin affinity of collagen/silk fibroin sponges can provide a wetting environment for wounds and reduce wound infiltration and bleeding. Importantly, by regulating the release of SCS in collagen/silk fibroin sponges, Mφs polarization towards the M2 phenotype can be regulated, thereby increasing the secretion of endogenous *VEGF*, mediating neovascularization and ultimately promoting late vascular maturation, granulation tissue growth and wound repair function. Our research can provide a new strategy for the clinical treatment of diabetes dressings.

## 2. Materials and Methods

### 2.1. Materials

Chitosan (deacetylation degree ≥95%, viscosity from 100 to 200 mpa.s.), sodium-hydroxide, chlorosulfonic acid, lithium bromide, glycidyl methacrylate, acetic acid and anhydrous ethanol were obtained from Aladdin (Wuhan, China). N-N dimethylformamide, anhydrous sodium carbonate and glutaraldehyde were purchased from Macklin (Shanghai, China). Dulbecco’s modified Eagle medium (DMEM), penicillin–streptomycin, fetal bovine serum (FBS) and phosphate buffer saline (PBS) were supplied by Gibco (Grand Island, NY, USA). A live/dead cell double-staining kit was obtained from Proteintech (Rosemont, IL, USA), and a cell counting kit-8 was purchased from KeyGen Biotech (Nanjing, China). Enzyme-linked immunosorbent assay kits were obtained from 4A Biotech (Beijing, China). Streptozotocin was obtained from Sigma Aldrich (Burlington, MA, USA). Citric acid was purchased from Solarbio (Beijing, China). Sodium citrate was supplied by YuanYe Biotech (Shanghai, China). A hematoxylin and eosin dying kit was purchased from Beyotime (Shanghai, China), and Masson’s Trichrome dying kit was purchased from Solabio (Beijing, China), dying according to the procedure in the instructions.

### 2.2. Synthesis of 2-N,6-O-Sulfonated Chitosan (26SCS)

The synthesis of 26SCS was performed with a slight modification of a previously described protocol. First, the sulfonation reagent was prepared by adding 10 mL HClSO_3_ into 30 mL N-N dimethylformamide (DMF), which was cooled at 4 °C in advance and stirred for 15 min. Secondly, the sulfonation reagent was transferred to a three-necked, flat-bottomed flask containing chitosan solution (2 g chitosan dispersed in 50 mL N-N dimethylformamide and 10 mL formic acid) and reacted at 50 °C for 3 h to obtain a light-yellow solution.

After the reaction was completed, the following post processing was performed. First, 100 mL deionized water was added to the above light-yellow solution to stop the reaction, and 1 M NaOH was used to adjust the pH to 7–8; then, 1500 mL anhydrous ethanol was added to precipitate product. The extracted product was dissolved in deionized water; then, the solution was dialyzed against water for 3–7 d in a 2000 Da cut-off dialysis membrane. Finally, the 26SCS was obtained after freeze-drying.

### 2.3. Preparation of SilMA

First, the natural silk was boiled with a 0.25% Na_2_CO_3_ aqueous solution for 30 min to remove sericin, and the step was repeated 3 times; then, the silk was dried in a 60 °C oven. Subsequently, the 10 g dried silk was dissolved in 50 mL lithium bromide solution (9.2 M); then, 424 mM glycidyl methacrylate (GMA) solution was added to the solution and reacted at 60 °C for 5 h. Finally, impurities were filtered from the solution, and the filtrate was dialyzed against water for 3–7 d in a 8000–14,000 Da cut-off dialysis membrane, and the water-soluble silk (SilMA) was obtained after lyophilization.

### 2.4. Preparation of 26SCS-Loaded Composite Sponges

First, 0.05 g collagen and 0.05 g SilMA were dissolved in 5 mL acetic acid (0.5 M) and mixed evenly; then different concentrations of 26SCS (2.5 mg/mL, 5 mg/mL, 7.5 mg/mL and 10 mg/mL) were added, and a uniform mixture was obtained after full stirring. The mixed solution was injected into the polytetrafluoroethylene mold, and a one-way temperature gradient with bottom-up temperature difference was set to form directional crystallization of the mixed solution, then freeze-dried. The obtained composite sponges were cross-linked in a glutaraldehyde atmosphere for 5 h. Finally, the composite sponges were immersed in anhydrous ethanol for 1 h and soaked in deionized water for 3 h. The 26SCS-loaded composite sponges were obtained after lyophilization ((2.5/5.0/7.5/10.0) 26SCS-loaded composite sponges).

### 2.5. Characterization Methods

#### 2.5.1. Fourier Transform Infrared Spectroscopy (FTIR)

The chemical structure changes of 26SCS and SilMA were recorded using a Fourier transform infrared spectrometer (Spectrum Two, PerkinElmer, Shelton, CT, USA) in the wavenumber range of 3500–500 cm^−1^ with a resolution of 4 cm^−1^.

#### 2.5.2. Nuclear Magnetic Resonance Spectroscopy (NMR)

The changes in functional groups before and after modification of chitosan and silk were recorded by nuclear magnetic resonance (AVANCE III 300, BRUKER, Ettlingen, Germany).

#### 2.5.3. Scanning Electron Microscopy (SEM)

The morphology and the layer spacing of composite sponges were characterized by SEM (LEO153VP, Carl Zeiss, Jena, Germany). The samples were pasted to the copper platform and gilded and sputtered; then, their cross-sectional/longitudinal morphologies were observed. SEM images of composite sponges with a magnification of 500× were captured; then, 150 holes were randomly selected to calculate the layer spacing using ImageJ (x64) 1.8.0. (National Institute of Health, Bethesda, MD, USA).

#### 2.5.4. Measurement of Porosity

The porosity of the composite sponges was determined by the ethanol immersion method. Briefly, the sponges were immersed in anhydrous ethanol under a vacuum atmosphere to ensure that the anhydrous ethanol fully permeated into the sponges. The wet weight (*W*_w_) of the composite sponges were measured after 24 h, and the porosity (*P*(%)) was defined as follows:

(1) $$P\left(\%\right)=\frac{\left(W_{d}-W_{w}\right)}{\rho V}\times 100\%$$

where *ρ* is the density of anhydrous ethanol, and *W*_d_ is the dry weight of the composite sponges.

#### 2.5.5. Measurement of Water Absorption

The fully dried composite sponges were weighed (*M*_0_), then immersed in DI water. Filter paper was used to remove the water adsorbed on the surface of the sponges, and the wet weight (*M*_1_) was recorded. The water absorption (*W*(%)) was defined as follows:

(2) $$W\left(\%\right)=\frac{\left(M_{1}-M_{0}\right)}{M_{0}}\times 100\%$$

#### 2.5.6. Measurement of the Release of 26SCS

The 26SCS-loaded composite sponges were immersed in centrifuge tubes containing PBS solution and incubated at 37 °C in a shaker with a speed of 90 r/min. The supernatant was withdrawn at different time points (1–7 days) for detection [37]. The absorbance of each sample was measured at a specific wavelength; then, the cumulative release of 26SCS was calculated according to the standard curve. Three parallel experiments were performed on each sample.

### 2.6. In Vitro Cell Response to 26SCS-SilMA/Col Sponges

#### 2.6.1. Cell Culture

The fibroblasts (L929), macrophages (RAW264.7) and HUVECs were purchased from the American Type Culture Collection (ATCC). All cells were cultured in Dulbecco’s modified Eagle medium containing 1% penicillin and 10% fetal bovine serum at 37 °C in a 5% CO_2_ cell incubator [37]. The medium was updated every other day. In order to evaluate the effect of the 26SCS concentration on cell behaviors, we set six SCS concentrations, namely 0 mg/mL, 1 mg/mL, 2 mg/mL, 3 mg/mL, 4 mg/mL and 5 mg/mL (named (0) 26SCS, (1) 26SCS, (2) 26SCS, (3) 26SCS, (4) 26SCS and (5) 26SCS, respectively), to determine the appropriate concentration range for SCS that could have a positive effect on cell function.

#### 2.6.2. Cytotoxicity Assay

The fibroblasts (L929) were seeded in 48-well plates containing different concentrations of 26SCS or covered with (2.5/5.0/7.5/10.0) 26SCS-loaded sponges at a density of 2 × 10^4^ cells per well. After 1, 4 and 7 days of incubation, the medium containing 10% CCK-8 was substituted for the original medium; then, the absorbance at 450 nm was measured using an enzyme-linked immunosorbent assay plate reader (MK3, Thermo Fisher Scientific Ltd., Waltham, MA, USA).

#### 2.6.3. Cell Viability Assay

Similarly, the fibroblasts (L929) were seeded at a density of 2 × 10^4^ cells per well in 48-well plates covered with 26SCS-loaded composite sponges. After 48 h of incubation, the original medium was replaced with reagent for live/dead cell staining. A fluorescence microscope (XDY-2, Guangzhou Liss Optical Instrument Ltd., Guangzhou, China) was used to observe the distribution of live/dead cells.

#### 2.6.4. Quantitative Real-Time RT-PCR (qRT-PCR)

The macrophages (RAW264.7) were co-cultured with the (0/1/2/3/4/5) 26SCS in 6-well plates at a density of 5 × 10^4^ cells/well. After 1, 3 and 7 days of incubation, the expression of polarization-related genes in Mφs was evaluated by qRT-PCR. In brief, total RNA was extracted from Mφs using a TrizolReagent Kit (Invitrogen, Carlsbad, CA, USA). The extracted RNA was reverse-transcribed into DNA using a PrimeScript RT reagent kit (Takara, Maebashi, Japan) according to the manufacturer’s instructions. The mRNA expressions of *IL-1β*, *TNF-α*, *INOS*, *OSM* and *Arg-1* were quantized by a SYBR Green PCR Master Mix real-time PCR system, and glyceraldehyde 3-phosphate dehydrogenase (GAPDH) was used as the normalized steward gene. The forward and reverse sequences of each gene primer are shown in Table 1.

#### 2.6.5. Enzyme-Linked Immunosorbent Assay (ELISA)

The macrophages (RAW264.7) were seeded at a density of 2 × 10^4^ cells per well on 48-well plates covered with composite sponges, and SilMA/Col-330 was used as the control group. After 1, 3 and 7 days of incubation, supernatants were collected, and Mφ polarization related factors *TNF-α*, *IL-1β*, *TGF-β1* and *OSM*, as well as angiogenesis-related factors *VEGF* and *PDGF-BB*, were determined by enzyme-linked immunosorbent assay (ELISA).

#### 2.6.6. Cell Scratch Assay

Macrophages (RAW264.7) were seeded at a density of 5 × 10^4^ cells/well in 6-well plates coated with composite sponges. After 3 and 7 days of cultivation, the supernatant was taken and centrifuged at 12,000 r/min for 10 min to obtain conditioned medium CM (CM3/CM7-(0/2.5/5.0/7.5) 26SCS). Human umbilical vein endothelial cells (HUVECs) were seeded in 6-well plates at a density of 5 × 10^5^ cells/well and incubated for 24 h. After forming a confluent monolayer, the cell monolayer was longitudinally scratched with a 200 µL sterile plastic tip. CMs were added to incubate for 0, 6, 12 and 24 h, and cell migration was measured using an FRD-6C microscope (FRD-6C, Cossim, Beijing, China). Five random fields were collected in each sample for analysis, and ImageJ (x64) 1.8.0 (National Institute of Health, Bethesda, MD, USA) was used to evaluate the wound area.

#### 2.6.7. Tube Formation Assay

HUVECs were seeded on Matrigel at a density of 5 × 10^5^ cells/well and incubated with CMs for 4 h. Network branches and lengths were calculated using images collected by the Angiogenesis Analyzer of ImageJ software (National Institute of Health, Bethesda, MD, USA).

### 2.7. In Vivo Wound Healing Analyses

#### 2.7.1. Construction of Diabetic Rat Model

Male Sprague–Dawley rats (12 weeks old and 200–300 g weight) were purchased from Southern Medical University. All animal experiments involved in this study were authorized by the Laboratory Animal Ethics Committee of Jinan University, and all surgical operations were performed in compliance with national legal requirements for animal husbandry. Male Sprague–Dawley rats were fasted for 12 h, then intraperitoneally injected with 100 μL streptozotocin; the blank group was injected with the same dose of sodium citrate buffer. After 7 days, the rats’ fasting blood glucose levels were measured using a glucose meter. The diabetic rat model was considered to be successfully established when the blood glucose level exceeded 13.8 mmol/L.

#### 2.7.2. Establishment of Back Wound Model in Diabetic Rats

The streptozotocin-induced diabetic rats were operated on to establish a wound model, and the rats were randomly divided into six groups (five in each group). First, the rats were anesthetized by intraperitoneal injection of 0.15 mL 3% pentobarbital sodium. Then, the backs of all rats were shaved and sterilized, and a full-thickness oval skin wound model with a diameter of about 9 mm was built on the back of each rat. To prevent the rats from scratching the wound, the wound edges of the rats were treated with sterile erythromycin ointment. Finally, five groups of composite sponges ((0/2.5/5.0/7.5/10.0) 26SCS-SilMA/Col-330) were implanted into the wounds and wrapped with gauze. The blank group did not receive any treatment, and the wound was naturally repaired. Photos of the wounds were taken on days 1, 3, 7 and 14, and Image J was used to measure the wound area. The calculation formula for the wound healing rate (*W*%) was defined as follows:

(3) $$W\left(\%\right)=\frac{\left(S_{t}\right)}{S_{0}}\times 100\%$$

where *S*_t_ is the area of the wound at the corresponding time point, and *S*_0_ is the area of the initial wound.

#### 2.7.3. Histological Analysis

Wound tissue was collected on day 14, fixed with 4% paraformaldehyde and embedded in paraffin. The slides were stained with hematoxylin and eosin (H&E) or Masson’s trichrome; the stained sections were photographed under a microscope, and wound healing was observed.

#### 2.7.4. Immunofluorescent Staining and Immunohistochemical Staining

First, the paraffin sections of wound tissue were dewaxed, rehydrated and boiled in sodium citrate antigen repair solution for 15 min. Then, the sections were treated with rabbit anti-CD86 (1:300, HUABIO, Hangzhou, China) and rabbit anti-CD206 (1:500, HUABIO, China), incubated at 4 °C overnight, washed with PBS and incubated at room temperature for 2 h with DyLight fluorescently labeled secondary antibodies (1:400, Abbkine, Wuhan, China). Finally, the nucleus was stained with an anti-quenching agent containing DAPI. The immunofluorescence tissue was observed and photographed using a laser confocal microscope (Zeiss, Oberkochen, Germany, LSM 800). For immunohistochemistry, the sections were treated with rabbit anti-CD31 (abcam, Cambridge, UK), and the stained slices were scanned using a scanner (VS200, OlyVIA, Tokyo, Japan). Five slices were used in each experimental group, and the total defect area of each slice was quantitatively analyzed.

### 2.8. Statistical Analysis

All the data are expressed as means ± standard deviation (SD), and all statistical analysis was carried out using SPSS version 10.1 software (SPSS Inc., Chicago, IL, USA). Statistically significant differences (*p* < 0.05) between the various groups were adjusted using the Tukey–Kramer post hoc test.

## 3. Results

### 3.1. Characterization of 2-N, 6-O-Sulfated Chitosan

**Figure 1:**
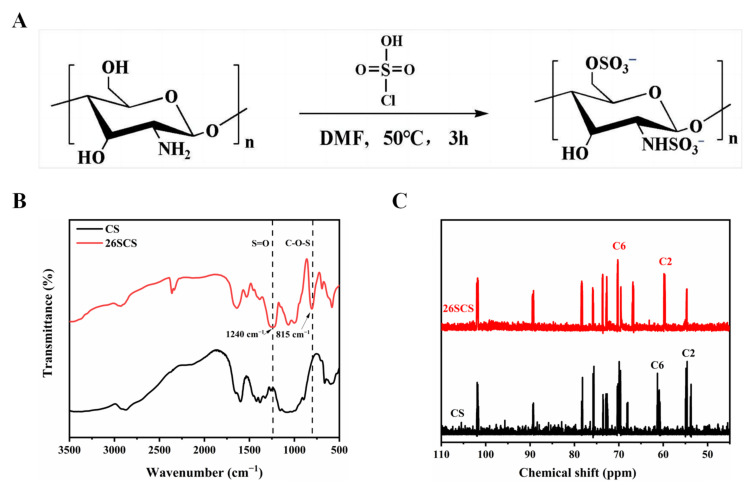
(**A**) Synthesis scheme of 26SCS. (**B**) The Fourier transform infrared spectra of 26SCS. (**C**) The carbon nuclear magnetic resonance spectra of 26SCS.

The synthesis scheme of 26SCS is shown in Figure 1A. The chemical structures of the synthesized products were recorded by Fourier transform infrared spectroscopy (FTIR) and carbon nuclear magnetic resonance (^13^C-NMR). There are three positions in the molecular structure of CS that may undergo sulfonation reaction, namely C2-NH_2_, C3-OH and C6-OH. Among them, the amino group at position C2 has the highest activity, followed by the primary hydroxyl group at position C6, so sulfonation reactions mainly occur at positions C2 and C6. Due to the low activity of the C3-hydroxyl group and the steric hindrance in the sulfonation reaction, the possibility of forming sulfonic groups at C3-OH is very minimal [39]. As shown in Figure 1B, the FTIR spectrum of 26SCS located at 1240 cm^−1^ and 815 cm^−1^ showed characteristic new absorptions due to the bond stretching of O=S=O and C-O-S, respectively. The ^13^C-NMR spectra showed that the absorption peaks of C-2 amino and C-6 hydroxyl groups of chitosan at 55 ppm and 61 ppm generated a chemical shift, moving to 60 ppm and 70 ppm, respectively, demonstrating the formation of new C2-SO_3_ and C6-SO_3_ absorption peaks, which also proved the successful sulfonation of chitosan [35].

### 3.2. Characterization of Methylallyl Acylated Silk Fibroin (SilMA)

**Figure 2:**
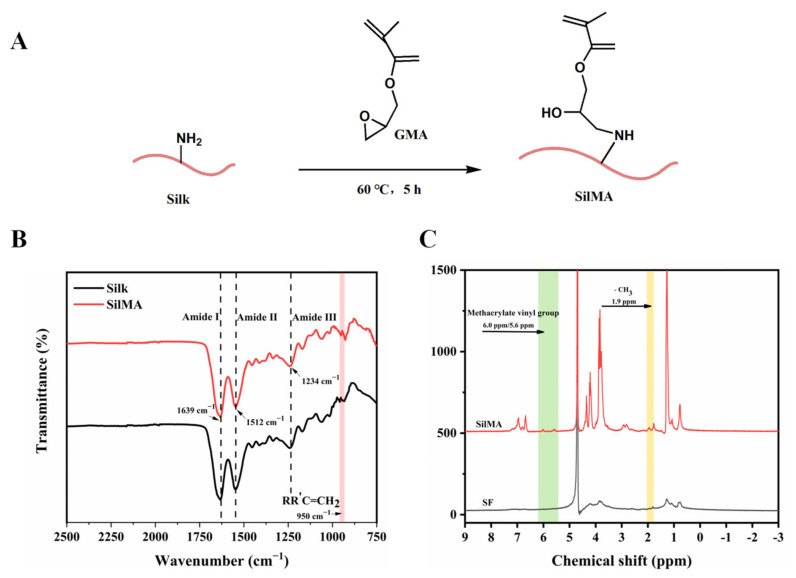
(**A**) Synthesis scheme of SilMA. (**B**) The Fourier transform infrared spectra of SilMA. (**C**) The proton nuclear magnetic resonance spectra of SilMA.

Due to the poor water solubility of natural silk, glycidyl methacrylate was used for modification to obtain a water-soluble SilMA, which facilitates the preparation of composite sponges in the later stage. The FTIR spectrum in Figure 2B shows that the synthesized product had three typical characteristic peaks at 1639 cm^−1^, 1512 cm^−1^ and 1234 cm^−1^, which belonged to the stretching vibrations of amide I, amide II and amide Ⅲ, respectively. Compared with the silk, the strength of the SilMA peak at 950 cm^−1^ was more pronounced due to the CH_2_ vibrational tensile peak of vinyl methacrylate. The GMA peak at 950 cm^−1^ was not obvious because the molecular weight of GMA is much smaller than that of SF, resulting few GMA functional groups that could be detected. Meanwhile, the characteristic resonances of methacrylate vinyl (at δ = 6.0 and 5.6 ppm) and the methyl group (δ= 1.9 ppm) were found by proton nuclear magnetic resonance (Figure 2C).

### 3.3. Characterization of Composite Sponges

The composite sponges were prepared using thermal phase separation combined with freeze-drying methods. Blending SilMA with Col to form composite sponges can not only fully utilize the good bio-compatibility and biological activity of Col but also improve its mechanical properties. In addition, a Schiff base reaction, which happened between the aldehyde groups of glutaraldehyde and the amino groups on SilMA or Col, was used to further improve the mechanical properties of the sponges. We first obtained SilMA/Col sponges with different pore sizes by adjusting the concentration of SilMA in the mixture during the preparation process, then selected the SilMA/Col sponges with a favorable comprehensive performance to prepare a series of 26SCS-loaded SilMA/Col sponges. In our previous work, SilMA/Col sponges with layer spacing of 330 ± 3.2 μm, 265 ± 4.8 μm, 125 ± 2.1 μm and 90 ± 1.5 μm were obtained by setting SilMA concentrations to 1%, 1.5%, 2% and 2.5%, respectively (named as SilMA/Col-330, SilMA/Col-265, SilMA/Col-125 and SilMA/Col-90, respectively) (Figure S1A). The porosity of the SilMA/Col-330, SilMA/Col-265, SilMA/Col-125 and SilMA/Col-90 was 94.37 ± 1.03%, 93.51 ± 1.23%, 83.71 ± 1.12% and 81.96 ± 1.62%, respectively (Figure S1B). SEM images showed that the SilMA/Col sponges presented a regularly arranged layered structure, with a large number of pores distributed in each layer, forming an interconnected pore structure (Figure S1C). The results of cell proliferation showed that L929 cells were able to proliferate in the composite sponges and maintain cell activity, demonstrating good cell compatibility (Figure S2A). The composite sponges with larger layer spacing exhibited faster cell proliferation rates and had a better effect in terms of promoting cytokine secretion (Figure S2B–F).

**Figure 3:**
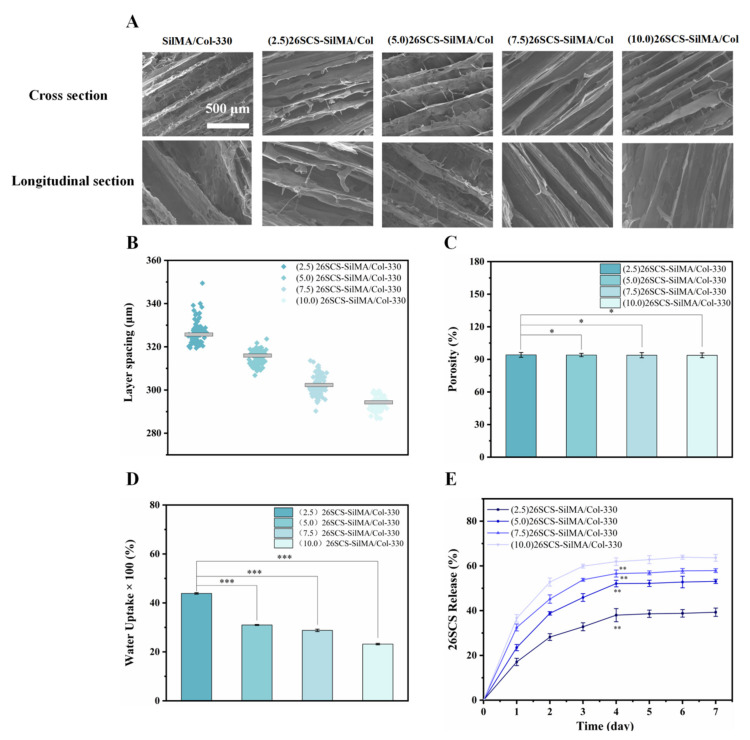
(**A**) SEM images of composite sponges loaded with 26SCS. (**B**) The layer spacing distribution of composite sponges. (**C**) The porosity of composite sponges. (**D**) The water absorption of composite sponges. (**E**) The determination of 26SCS release in composite sponges. * p < 0.05, ** p < 0.01, *** p < 0.001.

Considering all aspects of performance, we selected the SilMA/Col-330 loaded with 26SCS and prepared four groups of 26SCS-SilMA/Col-330 sponges. The loading concentrations of 26SCS were set to 2.5 mg/mL, 5.0 mg/mL, 7.5 mg/mL and 10.0 mg/mL, and the obtained 26SCS-loaded composite sponges were named (2.5) 26SCS-SilMA/Col-330, (5.0) 26SCS-SilMA/Col-330, (7.5) 26SCS-SilMA/Col-330 and (10.0) 26SCS-SilMA/Col-330, respectively. As shown in Figure 3A, all 26SCS-SilMA/Col-330 sponges maintained a neatly arranged layered structure (Figure S3A–E) with interconnected pores, which could ensure the smooth delivery of oxygen and nutrients during the co-culture process. The layer spacing of the (2.5/5.0/7.5/10.0) 26SCS-SilMA/Col-330 sponges were 325 ± 1.0 μm, 316 ± 0.8 μm, 302 ± 0.9 μm and 295 ± 1.2 μm (Figure 3B). Compared with the SilMA/Col-330, the layer spacing of the 26SCS-SilMA/Col-330 sponges appeared to be slightly decreased, and the higher the 26SCS loading concentration, the smaller the layer spacing. However, the porosity of the 26SCS-SilMA/Col-330 sponges maintained high porosity—all above 90%. Although the water absorption rate of the 26SCS-SilMA/Col-330 sponges decreased gradually with the increase in 26SCS loading concentration, the water absorption rates of all 26SCS-SilMA/Col-330 sponges exceeded 2000%, which could provide a moist environment for the wound and help to reduce the occurrence of bleeding.

### 3.4. Release of 26SCS

The release behavior of 26SCS-SilMA/Col-330 sponges was studied by measuring the release concentration of 26SCS in vitro and calculating the cumulative release amount at different time points. As shown in Figure 3E, the four groups of sponges showed rapid release of 26SCS in the first four days, then tended to be stable from days 4 to 7. The release concentration of 26SCS from the sponges gradually increased with increase in the loading concentration. The maximum release concentrations of the (2.5/5.0/7.5/10.0) 26SCS-SilMA/Col-330 sponges on day 4 were 0.2 ± 0.03 mg/mL, 1.09 ± 0.04 mg/mL, 2.36 ± 0.02 mg/mL and 3.93 ± 0.06 mg/mL.

### 3.5. Cytocompatibility of 26SCS and 26SCS-SilMA/Col-330 Sponges

**Figure 4:**
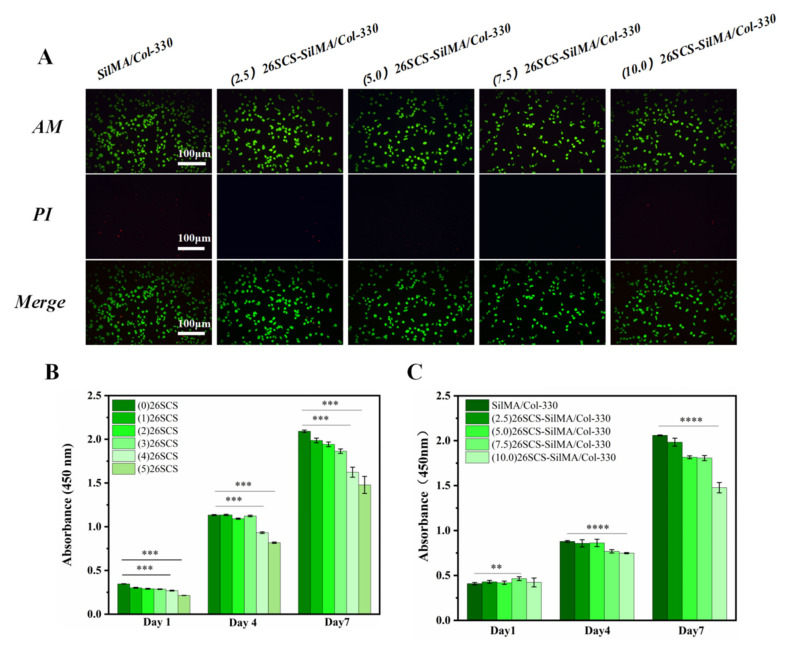
(**A**) Fluorescence microscopic image of L929 after co-culture for 48 h with composite sponge extract. (**B**) The cell proliferation of 26SCS. (**C**) The cell proliferation of composite sponges. * indicates significant differences between groups, * p < 0.05, ** p < 0.01, *** p < 0.001, **** *p* < 0.0001.

The cytotoxicity of 26SCS was first evaluated according to the grading criteria of cytotoxicity (Table S1). As shown in Figure 4B, when the concentration of 26SCS (*C_scs_*) was within the range of 0 < *C_scs_ ≤* 3 mg/mL, 26SCS showed no cytotoxicity to fibroblasts (L929) but showed mild cytotoxicity when the concentration of 26SCS exceeded 3 mg/mL. CCK-8 assay showed that the proliferation of L929 cells in 26SCS increased with the culture time. The proliferation rate of (1/2/3) 26SCS was slightly lower than that of the control ((0) 26SCS), while those of (4) 26SCS and (5) 26SCS were significantly lower than that of the control group, suggesting that 1 *≤ C_scs_≤* 3 mg/mL was an appropriate concentration range.

The cytocompatibility of the 26SCS-SilMA/Col-330 sponges was tested using a standard CCK-8 assay and a standard live/dead dye kit. Figure 4C shows that the cell proliferation rate in all 26SCS-SilMA/Col-330 sponges increased with the culture time. However, the cell proliferation rate in the (10.0) 26SCS-SilMA/Col-330 sponges was obviously lower than that in other groups, indicating that the (10.0) 26SCS-SilMA/Col-330 had a slight inhibitory effect on cell proliferation. This was because the 26SCS release concentration from the (10.0) 26SCS-SilMA/Col-330 reached 3.93 mg/mL on day 4, exceeding the appropriate range (1–3 mg/mL) and resulting in slight cytotoxicity and inhibition of the proliferation of L929. The fluorescent staining results in Figure 4A also show that the green fluorescence in the field of view gradually decreased with the increase in the concentration of loaded 26SCS. Therefore, it could be considered that the loading concentration of 26SCS in the 26SCS-SilMA/Col-330 sponges exceeded 10 mg/mL, which was not conducive to cell growth.

### 3.6. Effect of 26SCS on Cytokine Secretion of Mφs In Vitro (q-PCR)

**Figure 5:**
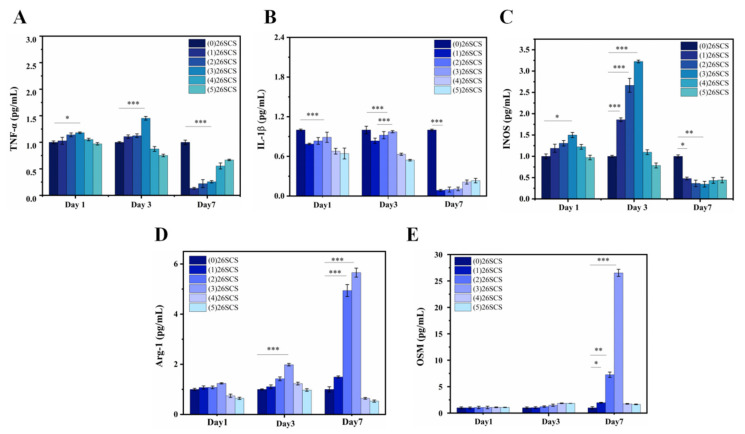
Mφ polarization-related gene expression in supernatant after 1, 3 and 7 days of co-culture with 26SCS. (**A**–**C**) The expression of pro-inflammatory genes TNF-ɑ, *IL-1β* and *INOS*. (**D**,**E**) The expression of anti-inflammatory genes *Arg-1* and *OSM*. * indicates significant differences between groups, * p < 0.05, ** p < 0.01, *** p <0.001.

In order to evaluate the effect of 26SCS concentration on Mφ polarization, macrophages were co-cultured with (1/2/3/4/5) 26SCS for 1, 3 and 7 days, and the expressions of polarization-related genes in Mφs were detected by qRT-PCR. Figure 5A–C show that compared with (0) 26SCS, the expressions of pro-inflammatory genes *TNF-β*, *IL-1β* and *INOS* in (1) 26SCS, (2) 26SCS and (3) 26SCS were up-regulated within 1–3 days and significantly down-regulated on day 7. The expression levels of M2-macrophage-related anti-inflammatory factors *Arg-1* and *OSM* in (1) 26SCS, (2) 26SCS and (3) 26SCS showed no significant difference within 1–3 days but were significantly up-regulated on day 7, especially in (2) 26SCS and (3) 26SCS, as shown in Figure 5D,E. The above results indicate that (1) 26SCS, (2) 26SCS and (3) 26SCS could initiate pro-inflammatory response in the initial stage of co-culture (1–3 days) and effectively down-regulate the expression of pro-inflammatory genes in the middle and later stages (3–7 days) to avoid excessive inflammation. In contrast, (4) 26SCS and (5) 26SCS did not play an important role in the regulation of the Mφ inflammatory response. We suggest that the optimal concentration range of 26SCS that could effectively regulate the Mφ polarization phenotype is 1–3 mg/mL.

### 3.7. Effect of 26SCS-SilMA/Col Sponges on Cytokine Secretion of Mφs In Vitro (ELISA)

**Figure 6:**
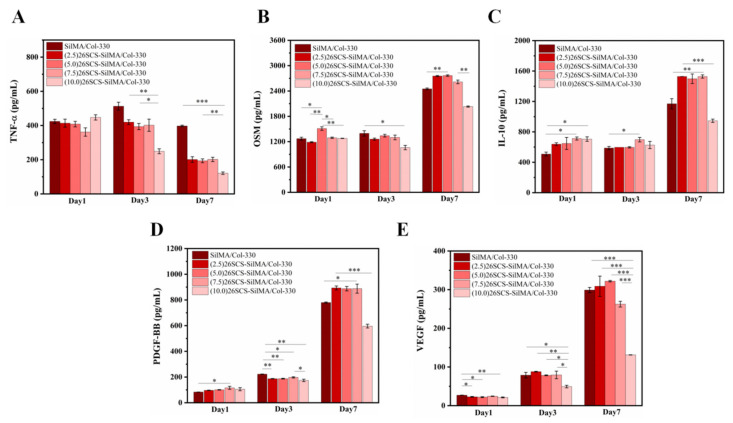
Mφ polarization-related cytokine secretion in the supernatant after 1, 3 and 7 days of co-culture with composite sponge scaffolds. (**A**) The secretion of the M1-macrophage-related factor TNF-ɑ. (**B**,**C**) The secretion of the M2-macrophage-related factors IL-10 and OSM. (**D**,**E**) The secretion of angiogenesis-related factors VEGF and PDGF-BB. * indicates significant differences between groups, * p < 0.05, ** p < 0.01, *** p < 0.001.

Figure 6A shows that *TNF-α* in the 26SCS-SilMA/Col-330 was activated in response to inflammation, up-regulated within 3 days of co-culture and significantly down-regulated on day 7, with a significant difference compared with the control group. The expression of IL-10 and OSM in the (2.5/5.0/7.5) 26SCS-SilMA/Col-330 sponges tended toward up-regulation and showed high levels when co-cultured for 3–7 days but appeared low in the (10.0) 26SCS-SilMA/Col-330 (lower than that in the control), as shown in Figure 6B,C. This was because the 26SCS released by the (10.0) 26SCS-SilMA/Col-330 sponges on day 3 reached 3.93 mg/mL, exceeding the appropriate concentration range, and produced certain cytotoxicity, thus affecting the phenotype polarization of macrophages. Figure 6E shows that the expression level of *VEGF* in both the 26SCS-SilMA/Col-330 groups and the control group (SilMA/Col-330) was low on day 1 and gradually up-regulated on day 3. However, *VEGF* was highly expressed in the (2.5/5.0/7.5) 26SCS-SilMA/Col-330 and the control on day 7. The expression level of *VEGF* in the (10.0) 26SCS-SilMA/Col-330 was significantly lower compared to other groups. Figure 6D shows that the expression of *PDGF-BB* in both the 26SCS-SilMA/Col-330 group and the control group (SilMA/Col-330) was at a low level on day 1 and gradually up-regulated with the culture time, showing a high level on day 7. Although the expression level of *PDGF-BB* in the 26SCS-SilMA/Col-330 was slightly lower than that in the control on day 3, it was significantly higher than that in the control on day 7 (except for (10.0) 26SCS-SilMA/Col-330). We suggest that the (10.0) 26SCS-SilMA/Col-330 not only could not regulate the secretion angiogenic factors *VEGF* and *PDGF-BB* by Mφs but might also have an inhibitory effect.

### 3.8. Angiogenesis In Vitro

**Figure 7:**
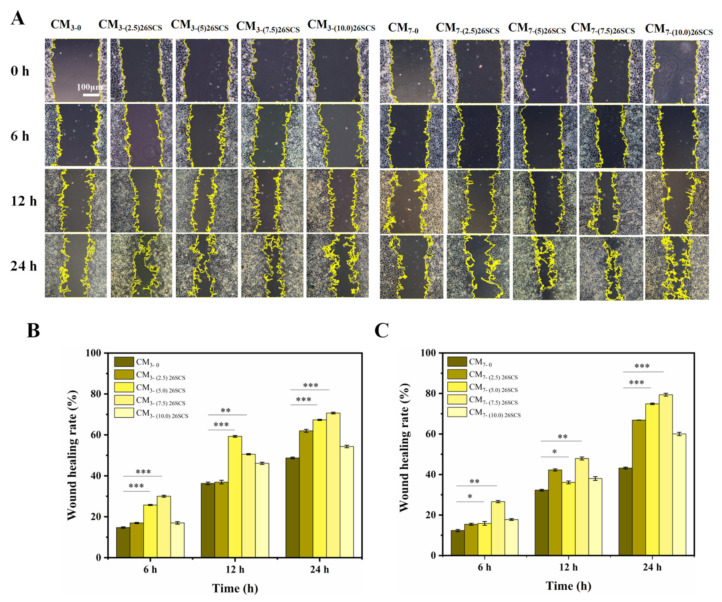
Representative photographs of cell migration after 0 h, 6 h, 12 h and 24 h of co-culture with conditioned medium. (**A**) Migration of HUVECs after co-culture with CM3 and CM7. (**B**) Quantitative analysis of the wound area after co-culture with CM3. (**C**) Quantitative analysis of the wound area after co-culture with CM7. * indicates significant differences between groups, * p < 0.05, ** p < 0.01, *** p < 0.001.

**Figure 8:**
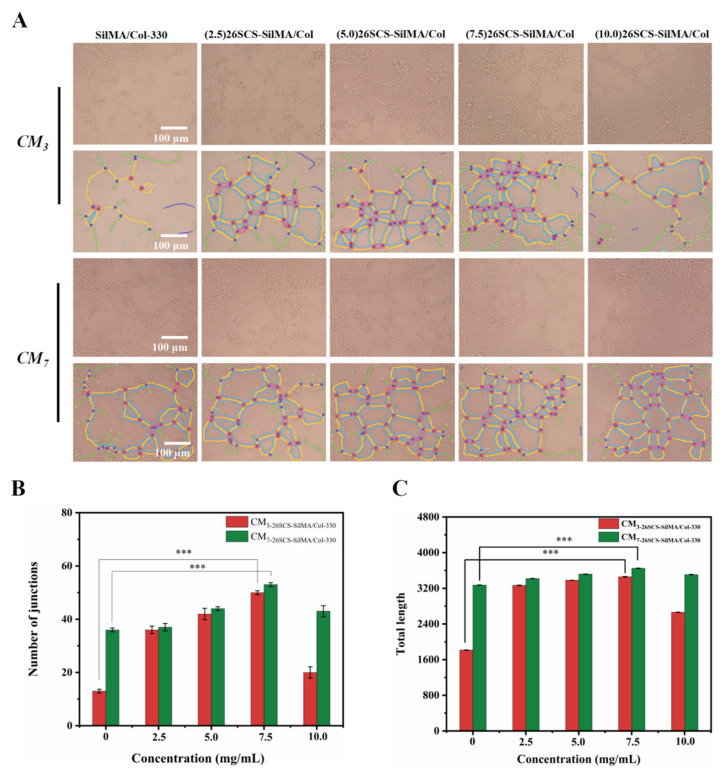
Representative photographs of tube formation after 4 h of co-culture with conditioned medium. (**A**) Tube formation of HUVECs after co-culture with CM3 and CM7. (**B**) Quantitative analysis of the junctions in tube formation. (**C**) Quantitative analysis of the total length in tube formation. * indicates significant differences between groups, * p < 0.05, ** p < 0.01, *** p < 0.001.

To estimate the influences of the 26SCS-SilMA/Col-330 sponges on angiogenesis in vitro, analyses of cell migration and tube formation activity of HUVECs were adopted. The conditioned media (CM) were first harvested from five groups of the sponges (the (2.5/5.0/7.5/10.0) 26SCS-SilMA/Col-330 and the SilMA/Col-330 (as the control)) and co-cultured with Mφs for 3 and 7 days (named CM3 and CM7, respectively), and HUVECs were incubated with CM for four hours to evaluate their migration capacity and tubular-forming activity. A scratch assay was used to determine the area of the scratch wound healing to assess the pro-migratory effects of the composite sponges on HUVECs. As shown in Figure 7, the scratch area of CM3/CM7-(0/2.5/5.0/7.5) 26SCS showed an obvious decreasing trend after 6, 12 and 24 h of culture, except for CM3/CM7-(10) 26SCS, and CM3/CM7-(5.0/7.5) 26SCS showed a smaller scratch area compared with other groups. The scratch area in CM7 appeared much smaller than that in CM3 after co-culture for 24 h, suggesting that CM7 exhibited an enhanced ability to induce migration of HUVECs and promote scratch healing. In addition, the results of Matrigel tube formation assays (see Figure 8 below) showed that the number of branch points and total capillary tube length of HUVECs with treatment of CM3/CM7-26SCS were higher than those in the control. HUVECs formed obvious capillary-like networks in CM3/CM7-26SCS, and CM7-(5.0/7.5) 26SCS showed an excellent tube formation promotion effect compared with other groups, while CM3/CM7-(10) 26SCS exhibited the weakest angiogenic capacity.

These findings demonstrated that the loading concentration of 26SCS in composite sponges and co-culture time had important effects on in vitro angiogenesis, which was closely related to the concentration of 26SCS released from the sponges during culture time. Figure 3E shows that 26SCS was rapidly released from sponges in the first four days, then manifested a stable liberation from days 4 to 7. The maximum release concentration of 26SCS from the (2.5/5.0/7.5) 26SCS-SilMA/Col-330 sponges was within the optimal concentration of 1–3 mg/mL, which provided favorable conditions for stimulation of Mφs to activate the M1 phenotype within 1–3 days, then transform into the M2 phenotype within 3–7 days. Moreover, the expression of angiogenic growth factors (*VEGF* and *PDGF-BB*) was significantly up-regulated on day 7, promoting the migration and angiogenesis of HUVECs. However, the maximum release concentration of 26SCS from the (10.0) 26SCS-SilMA/Col-330 was 3.93 mg/mL, which exceeded the optimal concentration range and failed to induce appropriate immune responses, leading to poor angiogenesis.

### 3.9. Analysis of Wound Healing In Vivo

**Figure 9:**
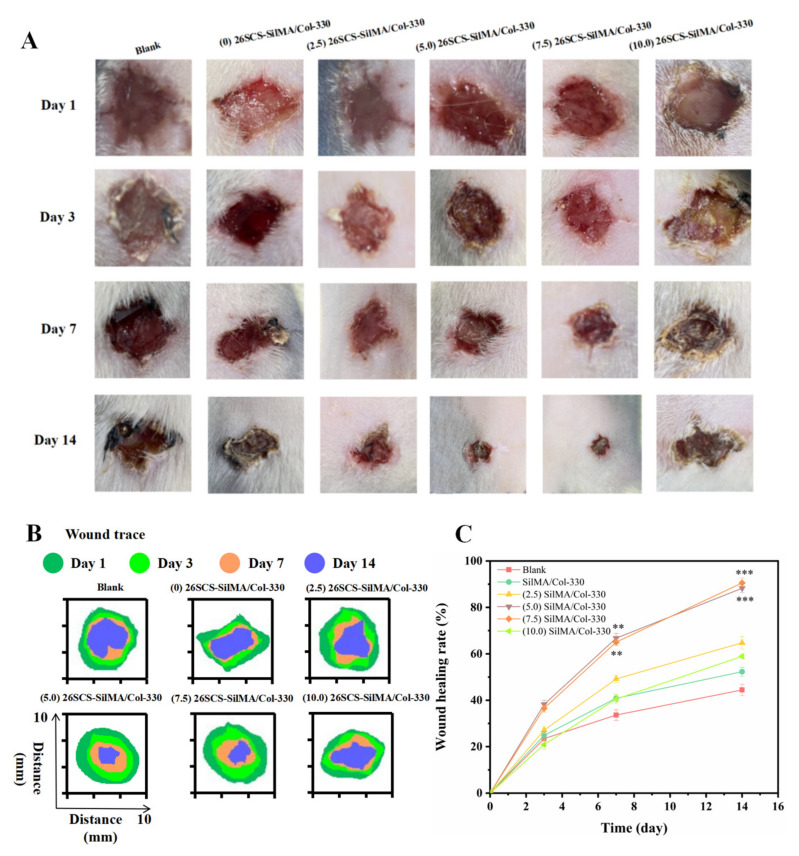
(**A**) Wound images after different treatments with sponges on days 1, 3, 7 and 14. (**B**) Wound healing was qualitatively analyzed using ImageJ. (**C**) Statistical wound healing rates after different treatments for varied time periods. * indicates significant differences between groups and blank group, * p < 0.05, ** p < 0.01, *** p < 0.001.

The in vivo wound healing performance of the 26SCS-SilMA/Col-330 sponges was evaluated in a full-thickness cutaneous wound model of diabetic rats. The wound area and healing rate were used to examine the repair effects in the different groups [40]. Figure 9 shows that the wound healing rates in all groups at different time points were gradually increased. On the 7th day, the wound healing rates in the (0/2.5/5/7.5/10) 26SCS-SilMA/Col-330 were 33.60 ± 2.28%, 40.88 ± 1.56%, 49.21 ± 1.56%, 66.74 ± 2.06% and 65.07 ± 1.39%. On the 14th day, the wound was almost completely healed in the (5.0) 26SCS-SilMA/Col-330 and (7.5) 26SCS-SilMA/Col-330-implanted area, and the wound areas were obviously reduced in the (2.5) 26SCS-SilMA/Col-330, indicating that the appropriate 26SCS release concentration could promote wound healing in diabetes rats. The 26SCS release from the (10.0) 26SCS-SilMA/Col-330 exceeded the appropriate concentration range, which might cause certain toxicity, resulting in limited positive effects and a delayed healing process, as well as scabs and some inflammatory cells at the wound site.

**Figure 10:**
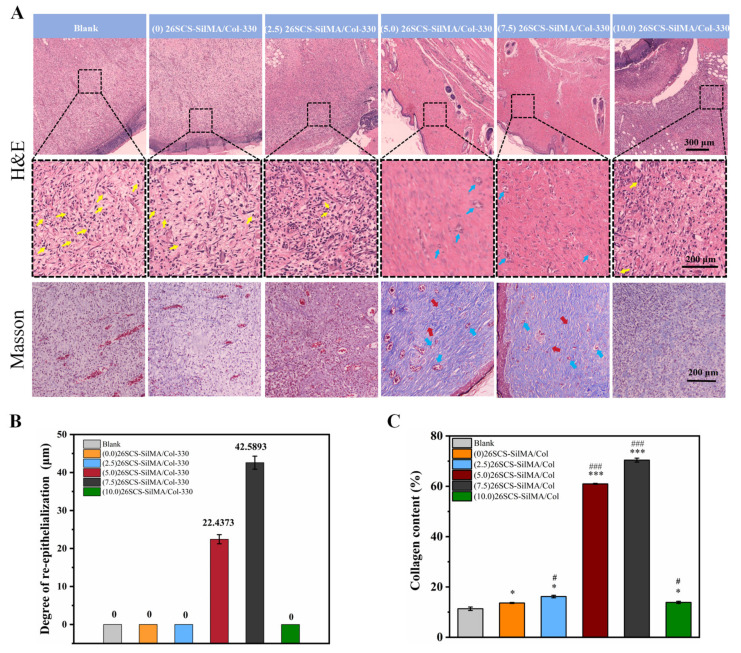
(**A**) H&E and Masson staining of the wound tissues on day 14. (**B**) Degree of re-epithelialization. (**C**) Collagen area quantitatively analyzed by ImageJ. Yellow arrows: neutrophils; blue arrows: neovascularization; red arrows: collagen fibers. * indicates significant difference between groups and blank group, * p < 0.05, ** p < 0.01, *** p <0.001; # represents a significant difference compared to the (0) 26SCS-SilMA/Col group; *# p*< 0.05, *## p*< 0.01, *### p* < 0.001.

Re-epithelialization of the wound epidermis, thicker granulation tissues, more collagen content and hair follicle regeneration can reflect wound healing. Histological analysis (Figure 10) including H&E and Masson staining revealed better epidermal recovery in the (5.0/7.5) 26SCS-SilMA/Col-330 groups, along with the formation of more abundant collagen deposition with aligned fibers in the skin tissue, as well as new hair follicles. Moreover, an increased number of blood vessels presented in the granulation tissue. In contrast, the epidermis wound site in the blank and (10) 26SCS-SilMA/Col-330 groups showed incomplete tissue, less collagen deposition and a lack of skin appendages such as hair follicles.

**Figure 11:**
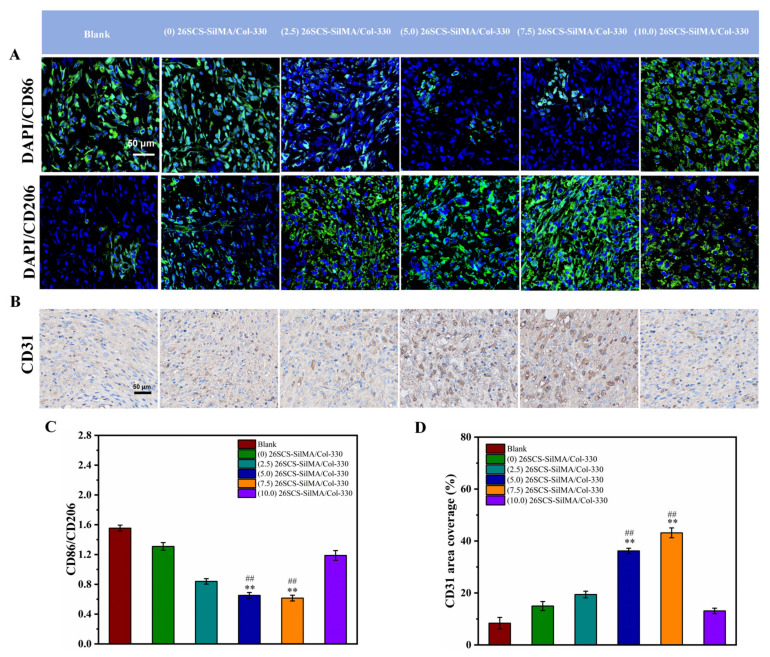
(**A**) Immunofluorescence staining of the wound tissues on day 14: CD86 and CD206. (**B**) Immunohistochemical staining of the wound tissues on day 14: CD31. (**C**) The CD86/CD206 ratio of the wound tissue. (**D**) Area coverage of CD31 of the wound tissue. * indicates significant difference between groups and blank group, * p < 0.05, ** p < 0.01, *** p <0.001; # represents a significant difference compared to the (0) 26SCS-SilMA/Col group; *# p* < 0.05, *## p* < 0.01, *### p* < 0.001.

Inflammation can prolong the process of wound healing. The macrophages associated with wound healing include CD86^+^ cells (M1 macrophages) and CD206^+^ cells (M2 macrophages), representing pro-inflammatory and anti-inflammatory phenotypes, respectively. The immunofluorescence staining and immunohistochemical staining analyses presented in Figure 11 show higher CD86 expression in the blank group, indicating a prolonged inflammation phase in diabetic wounds. In comparison, CD86 expression was lower and CD206 expression was higher in the (5.0/7.5) 26SCS-SilMA/Col-330 groups, indicating fewer inflammatory cell aggregations and inflammation alleviation. Figure 11 also shows that fewer neutrophils were present in the wound bed of (5.0/7.5) 26SCS-SilMA/Col-330 groups, while a large number of neutrophils was present in the blank group, suggesting that the release of 26SCS from 26SCS-SilMA/Col-330 might reverse the M1 phenotype by inducing Mφ polarization toward M2 subpopulations. Correspondingly, compared with the blank, the expressions of CD31 in the (5.0/7.5) 26SCS-SilMA/Col-330 groups were also higher, indicating that the composite sponges loaded with an appropriate concentration of 26SCS were conducive to the formation of blood vessels around the wound.

## 4. Discussion

As is well known, macrophages play an essential role in driving wound inflammatory response [40]. The abnormality of immune cells in diabetic wounds makes it difficult to suppress inflammation and keep the normal vascular system in a static state. To address this concern, we developed a bio-mimetic composite sponge dressing containing collagen and silk fibroin loaded with heparin-like polysaccharide (2-N, 6-O-sulfated chitosan), aiming to regulating the immune response of Mφs and prevent further M1 Mφ polarization, thus inducing effective angiogenesis in diabetic wounds.

The 26SCS-SilMA/Col-330 composite sponges prepared in this work exhibited a well-aligned lamellar structure with high porosity (more than 90%) and suitable layer spacing (295–325 μm), which not only provided active sites for cell adhesion and a cell-friendly micro-environment to promote cell infiltration but also provided mechanical support for the repair of damaged tissue [41]. The water uptake of all 26SCS-loaded composite sponges exceeded 2000%, indicating an excellent absorptive property conducive to maintaining the humidity of the wound [40].

Adequate vascular growth is critical in the healing process of diabetic wounds. In this work, the loading of 26SCS in the SilMA/Col sponges was designed to effectively induce angiogenesis by regulating the immune response of Mφs to up-regulate the expression of angiogenesis-related genes. It was found that the concentration of 26SCS had a significant impact on cell activity and Mφ phenotypic polarization. The concentration of 26SCS (*C_scs_*) was only within the range of 1 *≤ C_scs_≤* 3 mg/mL ((1)26SCS, (2)26SCS and (3)26SCS), L929 cells showed good cell compatibility and Mφs could guide appropriate inflammatory responses. Accordingly, we prepared four groups of 26SCS-loaded composite sponges by setting four different loading concentrations of 26SCS in SilMA/Col-330 sponges ((2.5/5.0/7.5/10.0) 26SCS-SilMA/Col-330) and studied the release behavior of 26SCS and the correlation between 26SCS release concentration, the Mφ polarization phenotype and angiogenesis.

The release profile shown in Figure 3E indicates that 26SCS presented rapid release from the composite sponges in the initial stage (1–3 days) and basically maintained stability on day 4. The maximum release concentration of 26SCS in the (2.5/5.0/7.5) 26SCS-SilMA/Col-330 was within the rage of 1–3 mg/mL (Figure 3E), while it was as high as 3.94 mg/mL in the (10.0) 26SCS-SilMA/Col-330, exceeding the appropriate range. The results of co-culture showed that both individual 26SCS medium and 26SCS-loaded composite sponges could induce M1/M2 phenotypic polarization and regulate the phenotypic transformation of Mφs. (1/2/3) 26SCS could effectively transform the Mφ phenotype from M1 to M2 by up-regulating the expression of pro-inflammatory gene TNF-α within 1–3 days and dramatically down-regulating the expression of *TNF-α* while up-regulating the expressions of anti-inflammatory genes *OSM* and *IL-10* on day 7. The trend of the expression level of pro-inflammatory gene *TNF-α* in the SilMA/Col-330 and the 26SCS-SilMA/Col-330 was similar to that in 26SCS. However, the expression of *TNF-α* in the 26SCS-SilMA/Col-330 was much lower than that in the SilMA/Col-330 during culture time. In particular, *TNF-α* expression was significantly down-regulated in the 26SCS-SilMA/Col-330 on day 7, while a higher expression level was maintained in the SilMA/Col-330. Moreover, the expressions of anti-inflammatory genes *OSM* and *IL-10* were dramatically up-regulated in the 26SCS-SilMA/Col-330 on day 7—much higher than that in the SilMA/Col-330. These findings indicate that the topological structure (porous and aligned lamellar structure) of composite sponges mainly triggered the pro-inflammatory response of Mφs in the early stage, leading to high expression of pro-inflammatory cytokine *TNF-α*. With the release of 26SCS from the sponges, the pro-inflammatory phenotypes of Mφs were gradually suppressed and tended towards an anti-inflammatory phenotype. We suggest that loading 26SCS onto the SilMA/Col sponges not only enhanced the biological activity of the sponges, providing a friendly micro-environment for cells through sustainable release of 26SCS, but also enabled a synergistic effect between sponges and 26SCS to induce a favorable immune response. The results of co-culture reveal that 26SCS-SilMA/Col-330 sponges exhibited a prominent advantage over SilMA/Col-330 in regulating Mφ polarization to M2 and stimulating secretion of two typical angiogenic growth factors, namely *VEGF* and *PDGF-BB*. As shown in Figure 6, the pro-inflammatory reaction of Mφs was initiated within 3 days, M1 Mφs were shifted to the M2 phenotype within 3 –7 days and the expressions of *VEGF* and *PDGF-BB* were significantly up-regulated on day 7, indicating that 26SCS-SilMA/Col-330 sponges could shift Mφ polarization to M2 in a timely manner to avoid excessive inflammation and elevated secretion of two typical angiogenic growth factors. Notably, the expressions levels of pro-inflammatory and anti-inflammatory factors, as well as *VEGF* and *PDGF-BB*, appeared higher in the (5.0/7.5) 26SCS-SilMA/Col-330 at different inflammatory stages, suggesting that 5 mg/mL and 7.5 mg/mL were the optimal loading concentrations of 26SCS to induce a positive inflammatory response. Correspondingly, CM7-(5.0/7.5) 26SCS exhibited a stronger vascularization ability than other groups. As shown in Figure 7 and Figure 8, the CM7-(5/7.5) 26SCS obviously promoted the migration speed of HUVECs and wound recovery and formed more capillary-like networks, more branches and longer tubes. In vivo data also revealed that 14 days post implantation, the (5.0/7.5) 26SCS SilMA/Col-330 groups exhibited fewer inflammatory cell aggregations, inflammation alleviation and faster re-epithelialization and formed more abundant collagen deposition, new hair follicles and sebaceous glands in the skin tissue. In addition, the granulation tissue implanted with the (5.0/7.5) 26SCS-SilMA/Col-330 showed a greater number of blood vessels than other groups.

We suggest that 26SCS efficiently induced angiogenesis by directing the immune response of Mφs with VEGF secretion, which is mainly attributed to the combined effect of sulfated amino groups (C2-NHSO^3−^) and the polysaccharide chain structure. It has been reported that the polysaccharide chain structure in heparin can provide biological effects. Heparin can interact with *VEGF* through its heparin-binding domain in the molecular structure and protect the biological activity of *VEGF*. The 26SCS synthesized in this work was a heparin-like anionic polysaccharide with sulfated amino groups and a polysaccharide chain structure. Incorporation of 26SCS into the SilMA/Col-330 endowed the composite sponges with special bio-functions. As shown in Figure 3E and Figure 6, a large amount of 26SCS was released from the sponges around the 4th day, promoting the secretion of anti-inflammatory cytokines *IL-10* and *OSM*, achieving timely transformation of Mφs from the M1 to M2 phenotype and preventing further polarization of M1-Mφ (the 7th day). Meanwhile, M2-Mφs secreted a large amount of angiogenic growth factors, and the 26SCS maintained the *VEGF* activity via the heparin-binding domain, promoting angiogenesis of HUVECs.

Mφ polarization is known to be mediated by canonical signal transducers and activators of transcription (Stat) signaling pathways [42]. The typical Stat signaling pathway is activated by *IL-4* or *IL-10* and tilts towards the M2 phenotype through the Stat6 or Stat3 pathway [42]. OSM is a multifunctional cytokine [43] that regulates many physiological processes in healthy individuals, such as by directly stimulating endothelial cells to influence the evolution of inflammatory responses [44] and contributing to tissue remodeling processes (Tanaka and Miyajima, 2003) [43]. The binding of active OSM to its receptor induces the phosphorylation of STAT3 (Fossey et al., 2011) [45], inducing signaling through the JAK–STAT pathway (West, et al.) [46]. In line with this knowledge, we proposed a possible mechanism by which 26SCS-SilMA/Col-330 sponges regulated Mφ polarization and promoted angiogenesis. In the first stage (1–3 days), macrophages, stimulated by physical sponge cues, were polarized into the M1 phenotype by secreting pro-inflammatory gene *TNF-α*, which initiated the inflammatory response [38,40]. With the release of 26SCS from sponges and the increase in its concentration, *TNF-α* expression was down-regulated, while anti-inflammatory cytokines *IL-10* and *OSM* were highly expressed, which might activate the phosphorylation of Stat3 in Mφs [34], inducing signaling through the Stat3 pathway [47]. Accordingly, Mφs promptly converted the M1 to the M2 phenotype in the second stage (3–7 days), secreting anti-inflammatory cytokines and inhibiting the further development of inflammation. Highly expressed *IL-10* and *OSM* stimulated Mφs to secrete a large amount of *VEGF* and *PDGF-BB*, promoting endothelial cell migration and inducing angiogenesis.

Here, we suggested that the regulation of inflammatory response and timely transition from the M1 to M2 phenotype could be achieved by regulating the release concentration and the release profile of 2626SC. The 26SCS-SilMA/Col-330 sponges without the addition of exogenous growth factors that we prepared in this work could become a bio-active material for the treatment of some inflammatory diseases.

## 5. Conclusions

In this work, we successfully prepared novel 26SCS-SilMA/Col-330 composite sponges with a well-aligned lamellar structure and high porosity and established a controlled-release system capable of delivering 26SCS for a long time. The highly aligned lamellar structure in the composite sponges exerted a “contact guidance” effect [48], guiding cell arrangement and migration. The concentration of 26SCS played an important role in inducing angiogenesis by directing the immune response of Mφs with VEGF and PDGF-BB secretion. The concentration of 26SCS within 1–3 mg/mL could effectively trigger an appropriate immune response, induce M1/M2 phenotypic polarization and regulate the phenotypic transformation of Mφs. The (5.0) 26SCS-SilMA/Col-330 and (7.5) 26SCS-SilMA/Col-330 sponges showed optimal biological activity, creating a favorable micro-environment for Mφs to guide the early M1 phenotype and the middle-to-late M2 phenotype, thereby up-regulating the expression of angiogenesis-related genes to promote angiogenesis. This in vivo study further demonstrated that the (5.0) 26SCS-SilMA/Col-330 and (7.5) 26SCS-SilMA/Col-330 sponges significantly improved neo-vascularization, re-epithelialization and collagen deposition while reducing inflammation in diabetic wounds bed and eventually accelerating wound healing. The 26SCS-SilMA/Col-330 sponges might provide a new approach to locally deliver 26SCS efficiently and hold great potential as novel dressings for the treatment of diabetic wounds.

## Figures and Tables

**Table 1:** Primer sequences used for qRT-PCR extraction.

Gene	Primer
*GAPDH*	Forward: GCAAGGATACTGAGAGCAAGAG
	Reverse: GGATGGAATTGTGAGGGAGATG
*IL-1β*	Forward: TGCCACCTTTTGACAGTGATG
	Reverse: TGATACTGCCTGCCTGAAGC
*TNF-* *ɑ*	Forward: GGCAGGTCTACTTTGGAGTCATTGC
	Reverse: ACATTCGAGGCTCCAGTGAATTCGG
*INOS*	Forward: AGCACAGAATGTTCCAGAATCCC
	Reverse: GTGAAATCCGATGTGGCCTTG
*OSM*	Forward: CACTCCTGTTTCCAAGCATG
	Reverse: CACGGGCACCCAGCATGGGG

## Data Availability

Data are contained within the article.

## Supplementary Materials

The following supporting information can be downloaded at: https://www.mdpi.com/article/10.3390/molecules29081832/s1, Figure S1: (A) The layer spacing of sponges. (B) The porosity of sponges detected by ethanol immersion method. (C) The SEM images of SilMA/Col sponges. * *p* < 0.05, ** *p* < 0.01, *** *p* < 0.001. Figure S2: (A) The cell proliferation of SilMA/Col sponges. (B)~(C) The expression of pro-inflammatory genes *IL-1β*, *TNF-ɑ*. (D)~(E) The expression of anti-inflammatory genes *OSM* and *TNF-β*. (F) The expression of angiogenesis gene *PDGF-BB*. * *p* < 0.05, ** *p* < 0.01, *** *p* < 0.001. Figure S3: The orientation of sponges with different 26SCS loads. Table S1: Cytotoxicity Grading Evaluation Criteria.
