# Supplementary material for: 26SCS-Loaded SilMA/Col Composite Sponge with Well-Arranged Layers Promotes Angiogenesis-Based Diabetic Wound Repair by Mediating Macrophage Inflammatory Response
Source: Molecules. 2024 Apr 17;29(8):1832. doi: 10.3390/molecules29081832 (PMC11053466; doi:10.3390/molecules29081832)
Supplement: Supplementary file 1 [file molecules-29-01832-s001.zip › molecules-2910712-supplementary.pdf]

Supporting Information

1. Supplementary Data

1.1 The layer spacing of SilMA/Col sponges

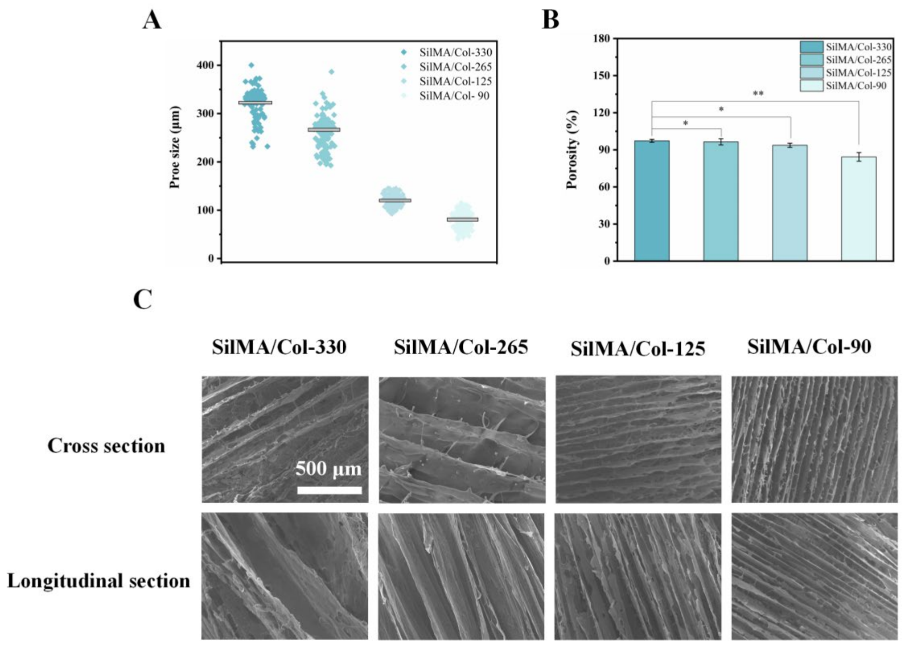

Figure S1. (A) The layer spacing of sponges. (B) The porosity of sponges detected by ethanol immersion method. (C) The SEM images of SilMA/Col sponges. **P<0.05*, ***P<0.01*, ****P<0.001*.

1.2 Data on co-culture of cell with SilMA/Col sponges

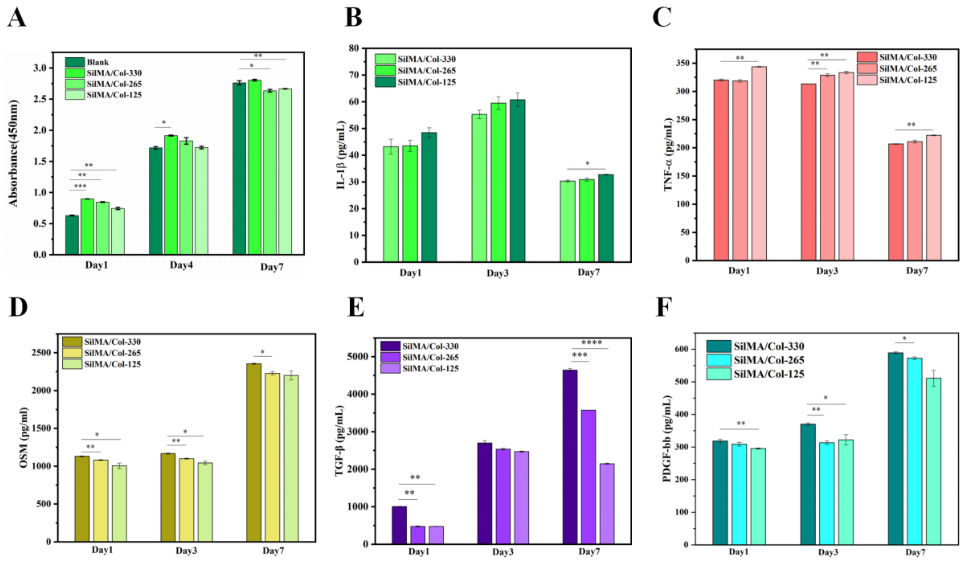

Figure S2. (A) The cell proliferation of SilMA/Col sponges. (B)~(C) The expression of

pro-inflammatory genes IL-1β, TNF-α. (D)~(E) The expression of anti-inflammatory genes OSM and TNF-β. (F) The expression of angiogenesis gene PDGF-BB. *$P<0.05$*, ***$P<0.01$*, ****$P<0.001$*.

1.3 Evaluation of fiber orientation in composite sponges

In order to characterize the fiber orientation in composite sponges, several regions were randomly selected and Image J was used to calculate the angle between the fiber orientation and the horizontal direction. The order parameter (S) is introduced to quantify the fiber orientation in sponges. An S value of 1 indicates that the fiber orientation in the sponge is completely consistent, and an S value of 0 indicates that the fiber orientation is completely disordered. The closer S is to 1, the higher the degree of orientation. The calculation method of ordered parameters is mainly referred to DOI: 10.27012 /, dc nki. Gdhuu. 2023.000077.

As could be seen from Fig.S3, the S of all PLGA-based fiber scaffolds was greater than 0.9, indicating that the fibers in the scaffolds were highly oriented and neatly arranged.

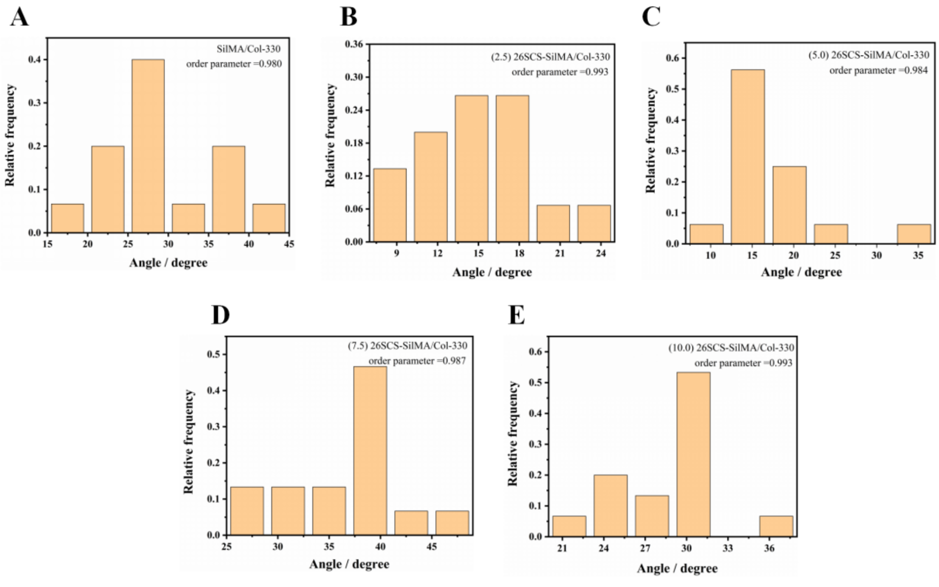

Fig.S3 The orientation of sponges with different 26SCS loads.

1.4 Cytotoxicity grading criteria

The cytotoxicity grading standards used for cytotoxicity evaluation are shown in Table.S1. RGR is calculated as follows:

$$RGR = \frac{A_1}{A_0} \times 100\%$$

where A_1 is absorbance of the experimental group after 48 h culture and A_0 is absorbance of control group after 48 h culture.

Table.S1 Cytotoxicity Grading Evaluation Criteria

RGR	Cytotoxicity （CTS）	Evaluation
75-99	I	None
50-74	II	Mild
25-49	III	Moderate
1-24	IV	Moderate
0	V	Severe
